# Supplementary material for: Recruiters’ perspectives and experiences of trial recruitment processes: a qualitative evidence synthesis protocol
Source: BMJ Open. 2021 Oct 22;11(10):e045233. doi: 10.1136/bmjopen-2020-045233 (PMC8543629; doi:10.1136/bmjopen-2020-045233)
Supplement: Supplementary data [file bmjopen-2020-045233supp001.pdf]

Ovid MEDLINE(R) and Epub Ahead of Print, In-Process & Other Non-Indexed Citations

1 exp Health Personnel/
2 clinician*.tw.
3 health professional.tw.
4 healthcare professional*.tw.
5 recruiter*.tw.
6 (health* adj2 professional*).tw.
7 Physicians/
8 Nurses/
9 Surgeons/
10 Research nurse.tw.
11 Investigator*.tw.
12 1 or 2 or 3 or 4 or 5 or 6 or 7 or 8 or 9 or 10 or 11
13 experience*.tw.
14 perceive.tw.
15 perception*.tw.
16 exp attitude/ or "attitude of health personnel"/
17 barrier*.tw.
18 facilitat*.tw.
19 challenge*.tw.
20 perspective*.tw.
21 belief*.tw.
22 opportunit*.tw.
23 opinion*.tw.
24 Decision*.tw.
25 view*.tw.
26 thought*.tw.
27 understand*.tw.
28 exp Decision Making/
29 Therapeutic Equipoise/
30 Equipoise.tw.
31 13 or 14 or 15 or 16 or 17 or 18 or 19 or 20 or 21 or 22 or 23 or 24 or 25 or 26 or 27 or 28 or 29 or 30
32 recruit*.tw.
33 participat*.tw.
34 enrol*.tw.
35 join*.tw.
36 select*.tw.
37 agree*.tw.
38 accept*.tw.
39 Informed Consent/
40 declin*.tw.
41 refuse*.tw.
42 refusal.tw.
43 decide.tw.
44 take part.tw.
45 accrual.tw.

46	exp Patient Selection/
47	32 or 33 or 34 or 35 or 36 or 37 or 38 or 39 or 40 or 41 or 42 or 43 or 44 or 45 or 46
48	Randomized Controlled Trials as Topic/
49	Clinical Trial/
50	exp clinical trial/ or exp clinical trials as Topic/
51	(RCT or RCTs).tw.
52	(random* adj3 trial).tw.
53	trial*.ti
54	control* trial.tw
55	48 or 49 or 50 or 51 or 52 or 53 or 54
56	qualitative research/
57	qualitative.tw.
58	ethnograph*.tw.
59	phenomenol*.tw.
60	grounded theory/
61	mixed method*.tw.
62	hermeneutic*.tw.
63	observation*.tw.
64	Focus Groups/
65	INTERVIEW/
66	semi-structured interview.tw.
67	Interviews as Topic/
68	56 or 57 or 58 or 59 or 60 or 61 or 62 or 63 or 64 or 65 or 66 or 67
69	12 and 31 and 47 and 55 and 68
